# Biohydrogenation Pathway of α-Linolenic Acid in Rumen of Dairy Cow In Vitro

**DOI:** 10.3390/ani12040502

**Published:** 2022-02-17

**Authors:** Guoxin Huang, Liya Guo, Meiqing Chen, Xufang Wu, Wenhao Tang, Nan Zheng, Shengguo Zhao, Yangdong Zhang, Jiaqi Wang

**Affiliations:** 1 Key Laboratory of Quality & Safety Control for Milk and Dairy Products of Ministry of Agriculture and Rural Affairs, Institute of Animal Sciences, Chinese Academy of Agricultural Sciences, Beijing 100193, China; huangguoxin1991@163.com (G.H.); mqchen1997@163.com (M.C.); wuxufang0502@163.com (X.W.); tangwenhao96@163.com (W.T.); zhengnan_1980@126.com (N.Z.); zhaoshengguo1984@163.com (S.Z.); 2 State Key Laboratory of Animal Nutrition, Institute of Animal Sciences, Chinese Academy of Agricultural Sciences, No. 2 Yuanmingyuan West Road, Beijing 100193, China; 3 College of Animal Sciences and Technology, Northeast Agricultural University, Harbin 150030, China; 4 Henan Institute of Science and Technology, Xinxiang 453003, China; gly2233@126.com

**Keywords:** α-Linolenic acid, *t*9*c*12*c*15-C18:3, rumen biohydrogenation, in vitro

## Abstract

**Simple Summary:**

Few studies have investigated a relationship between *t*9,*c*12,*c*15-C18:3 and ALA, an α-Linolenic acid *c*9,*c*12,*c*15-C18:3 in the rumen. These results indicated that *t*9,*c*12,*c*15-C18:3 was an intermediate of the α-linolenic acid shifted rumen biohydrogenation pathway. This study hypothesized a pathway for α-linolenic acid biohydrogenation in rumen.

**Abstract:**

The *t*9,*c*12,*c*15-C18:3 as an isomer of α-linolenic acid (*c*9,*c*12,*c*15-C18:3; ALA), has been recently detected in milk, but has not been found in the rumen. This study hypothesized that it may be a biohydrogenation product of ALA in rumen and aimed to explore whether it was present in the rumen and help to understand the rumen biohydrogenation mechanisms of ALA. The in vitro experiment included two treatments, a control check (CK group) with 50 µL ethanol added, and ALA group with 50 µL ethanol and 2.6 mg ALA (ALA addition calculated by 1.30% of dry matter base of diet); each sample of fermentation fluid had the composition of C18 fatty acids analyzed at 0, 0.5, 1, 2, 3, 4, 5, and 6 h. The results showed that no *t*9,*c*12,*c*15-C18:3 was detected in the CK group, but ALA addition increased the concentration of *t*9,*c*12,*c*15-C18:3 in fermentation fluid. The content of *t*9,*c*12,*c*15-C18:3 peaked 1 h after fermentation, then declined gradually. At 1 h, no *t*9*c*12*c*15-C18:3 was detected in the fermentation fluid of the CK treatment. The results suggested that ALA converted to the isomer *t*9,*c*12,*c*15-C18:3 through biohydrogenation in the rumen. The addition of ALA can also increase the concentration of *t*9,*c*12-C18:2, *c*9,*t*11-C18:2, *c*12-C18:1, *t*11-C18:1, *t*9-C18:1, and *c*6-C18:1 in fermentation fluid. It was concluded using an in vitro experiment that *t*9,*c*12,*c*15-C18:3 was a product of rumen biohydrogenation of ALA.

## 1. Introduction

The supplementation of α-linolenic acid (*c*9,*c*12,*c*15-C18:3; ALA) in the ruminant diet can increase the concentration of ALA and longer chain ω-3 polyunsaturated fatty acids (n-3 PUFA) in milk [1], which are essential fatty acids for humans. However, ALA biohydrogenation in the rumen is the main limiting factor influencing the efficiency of dietary ALA transport into milk. Therefore, exploring the pathways of ALA biohydrogenation in rumen could help regulate milk ALA and n-3 PUFA. The first step of ALA biohydrogenation in the rumen is isomerization. Pervious research showed that the *cis-trans* isomerization of ALA could happen in the ortho-position, and ALA could isomerize to *t*10,*c*12,*c*15-C18:3 with *c*9 to *t*10 [2], *c*9,*t*11,*c*15-C18:3 with *c*12 to *t*11 [3], and *c*9,*t*13,*c*15-C18:3 with *c*12 to *t*13 [4]. However, few studies have reported that the isomerization of ALA could happen in an in-situ position such as *t*9,*c*12,*c*15-C18:3 with *c*9 to *t*9 in the rumen. The in situ isomerization of ALA has been reported in the processing of food. A previous study reported that ALA could isomerize to *t*9,*c*12,*c*15-C18:3 through frying [5]. Recent studies have found that *t*9,*c*12,*c*15-C18:3 was present in milk [6]. Therefore, it can be hypothesized that ALA may also convert to *t*9,*c*12,*c*15-C18:3 through biohydrogenation in rumen. The purpose of this experiment was to explore whether there was *t*9,*c*12,*c*15-C18:3 in rumen fluid using an in vitro fermentation test and to investigate the relationship between *t*9,*c*12,*c*15-C18:3 and ALA. The *t*9,*c*12,*c*15-C18:3 identified in this study may provide a theoretical basis for the exploration of rumen biohydrogenation.

## 2. Materials and Methods

### 2.1. Experimental Design and Animal Management

The ALA pure products were purchased from Shanghai Yuanye Bio-Technology Co., Ltd. (Shanghai, China) and diluted with ethanol. Rumen fluid was collected from three ruminal cannulated Holstein cows fed a diet that met the feeding standards of dairy cattle in China according to the Ministry of Agriculture of China Feeding Standard of Dairy Cattle (NY/T 34-2004, MOA: Beijing, China, 2004). Rumen contents were withdrawn before the first meal in the morning, pooled in equal proportions into a container with CO_2_, transferred to the laboratory, and strained through four layers of cheesecloth to obtain rumen fluid.

Eighty fermentation bottles were separated into two groups as a control (CK group), with 50 µL ethanol added and ALA group with 50 µL ethanol and 2.6 mg ALA (ALA addition calculated by 1.30% of dry matter base of diet). The bottles were prewarmed to 39 °C and flushed by CO_2_, then filled with 200 mg diet (Table S1) and 30 mL oxygen-free buffered rumen fluid, including 10 mL rumen fluid and 20 mL buffer [7]. All the bottles were sealed with rubber stoppers and incubated in the shaking water bath (MRT-60R, shanghai minquan instrument Co., Ltd. Shanghai, China) at 40 r/min and 39 °C for 0, 0.5, 1, 2, 3, 4, 5, and 6 h, respectively. Fermentation fluid was collected in a 5 mL EP tube and stored at −80 °C for further C18 fatty acid analysis.

### 2.2. C18 Fatty Acid Analysis

The extraction of fatty acids and fatty acid methyl esters (FAMEs) from ruminal fluid was according to a previous protocol [6]. A 2 mL aliquot of each sample was transferred to a 15 mL tube and 4 mL of n-hexane/isopropanol (*v*/*v* = 3/2) solution was used to extract the fat. The methyl esterification of the fat was then performed using 10% acetyl chlorocarbinol followed by 2% methanolic NaOH. The FAMES were analyzed using an Agilent 7890A gas chromatograph (Agilent Technologies, Santa Clara, CA, USA) fitted to a 5975C MS mass spectrometer detector (Agilent Technologies, Santa Clara, CA, USA). A sample of 2 μL FAME mixed with hexane was injected through the split injection port in a 10:1 ratio into a capillary column CP-Sil 88 fused silica 100 m × 0.25 mm × 0.20 μm (Agilent, Palo Alto, CA, USA). The injector temperature was set at 250 °C, and oven temperature was initially 120 °C. After holding for 2 min, the temperature was increased by 3 °C per min to 180 °C and then increased by 1.5 °C per min to 200 °C. This temperature was maintained for three min, then increased by 2 °C per min to 220 °C and held for 20 min. The transfer line and MS ion source temperatures were maintained at 250 and 280 °C, respectively, and the ionizing energy was 70 eV.

The FAME peaks were identified using known FAME standards, including linoleic acid methyl ester mix CRM 47791 (*t*9,*t*12-C18:2, *c*9,*t*12-C18:2, *t*9,*c*12-C18:2 and *c*9,*c*12-C18:2), α-linolenic acid methyl ester mix CRM 47792 including (*t*9,*t*12,*t*15-C18:3, *t*9,*t*12,*c*15-C18:3, *t*9,*c*12,*t*15-C18:3, *c*6,*c*9,*c*12-C18:3, *c*9,*t*12,*t*15-C18:3, *c*9,*c*12,*t*15-C18:3, *c*9,*t*12,*c*15-C18:3, and *t*9,*c*12,*c*15-C18:3), CLA methyl ester mix Nu-Check prep including *c*9,*t*11-C18:2, *t*10,*c*12-C18:2 (Elysian, MN, USA), *c*6,*c*9,*c*12-C18:3, *t*6-C18:1, *t*9-C18:1, *t*11-C18:1, *c*6-C18:1, *c*9-C18:1, *c*11-C18:1, and C18:0 FAME standards individually from Nu-Check Prep (Elysian, MN, USA), *c*8-C18:1 and *c*12-C18:1 FAME individual standards from Cayman (Ann Arbor, MI, USA), *c*9-C18:1, *c*9,*c*12-C18:2 and *c*9,*c*12,*c*15-C18:3 triacylglycerols (TAGs) individual standards from Nu-Check Prep (Elysian, MN, USA), and *c*6,*c*9,*c*12-C18:3 TAGs from BePure (Beijing, China). The method of Chen et al. [6] was used to calculate fatty acids from FAME data.

### 2.3. Statistical Analysis

The results of C18 fatty acid were analyzed using ANOVA models of SAS (version 9.4, SAS Inst., Inc., Cary, NC, USA). The following statistical model was used:

Yij = μ + Ti + εij,(1)

where Yij represents the observed dependent variables, μ was the overall mean, Ti was the effect of treatment, and εij was the residual error. Significant and extremely significant levels were set at *p* < 0.05 and *p* < 0.01, respectively.

## 3. Results and Discussion

**Figure 1:**
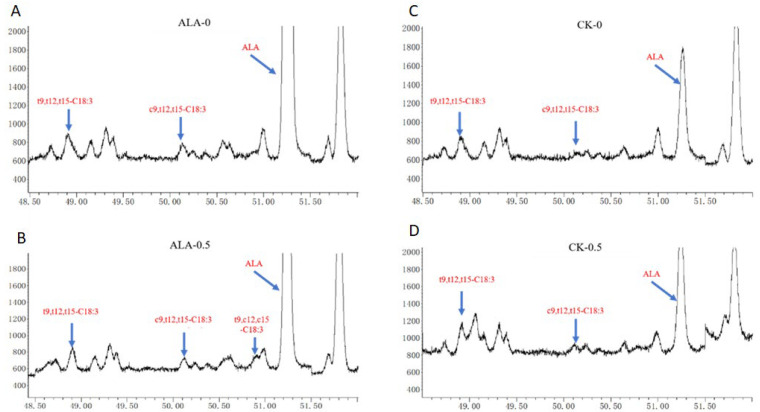
C18:3 fatty acid from fermentation in vitro. (**A**): ALA group fermented for 0 h; (**B**): ALA group fermented for 0.5 h; (**C**): CK group fermented for 0 h; (**D**): CK group fermented for 0.5 h.

The composition of C18 fatty acids detected in fermentation in vitro of the two groups is shown in Figure 1. The *t*9,*t*12,*c*15-C18:3, *t*9,*c*2,*t*15-C18:3, *c*6,*c*9,*c*12-C18:3, *c*9,*c*12,*t*15-C18:3 and *c*9,*t*12,*c*15-C18:3 were undetected in this experiment, and *t*9,*t*12,*t*15-C18:3, *c*9*t*12*t*15-C18:3, and ALA were detected in the CK and ALA groups. The *t*9,*c*12,*c*15-C18:3 was found in the ALA group after fermentation. Previous studies reported that *t*9,*c*12,*c*15-C18:3 has been found in the processing of food [5]. Recent studies have found that there are *t*9,*c*12,*c*15-C18:3 in milk [6], but the concentration was low. 

**Figure 2:**
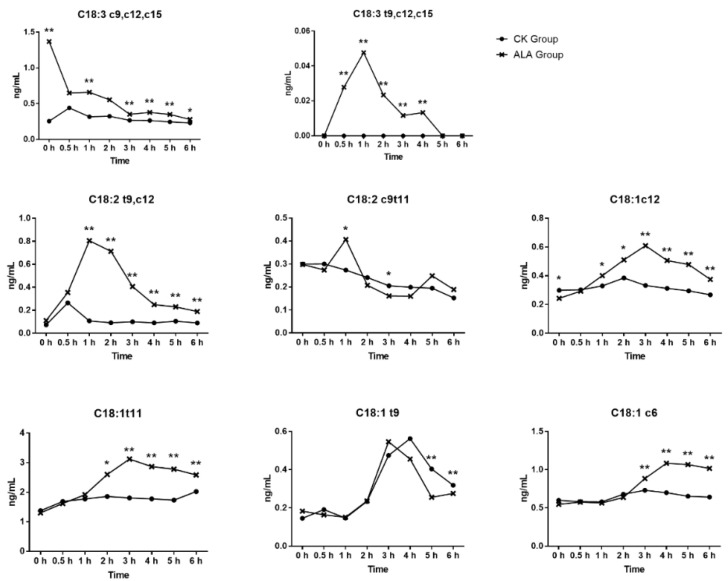
The C18 fatty acid content in fermentation in vitro at separate times. Significant difference: * *p* < 0.05, ** *p* < 0.01.

In this experiment, ALA influenced the content of C18 fatty acids in fermentation, as showed in Figure 2. The addition of ALA in fermentation increased the content of ALA in fluid from 0.256 ng/mL to 1.369 ng/mL (*p* < 0.01). With the extension of fermentation time, the content of ALA was decreased gradually, and the decline was the fastest in the first 0.5 h of fermentation. The concentration of *t*9,*c*12,*c*15-C18:3 decreased gradually with the fermentation time and the content of *t*9,*c*12,*c*15-C18:3 reached a peak 1 h after fermentation, then declined gradually. The addition of ALA increased the concentration of *t*9,*c*12-C18:2, *c*9,*t*11-C18:2, *c*12-C18:1, *t*11-C18:1, *t*9-C18:1, and *c*6-C18:1 in the fermentation fluid (*p* < 0.01). The change of *t*9*c*12-C18:2 showed the same trend, peaking 1 h after fermentation, then declining gradually. In this study, the change in *c*12-C18:1, *t*11-C18:1 showed a similar trend, the highest at 3 h after fermentation, then declined gradually and the concentration of *c*6-C18:1 increased at 3 h after fermentation. Through the biohydrogenation of ALA, the content of ALA decreased and converted to other C18 fatty acids [8]. Baldin et al. [9] reported that the content of ALA in fermentation fluid disappeared rapidly at the initial stages up to 0.5 h, which was like this study. The ALA isomers were further converted to C18:2 fatty acids [2], and the isomer *c*9,*t*11-C18:2 was one of the ALA biohydrogenation products present in the rumen. In the in vivo experiment, enhancing the content of ALA in the diet of cows also increased *c*9*,t*11-C18:2 in the rumen [10], the same result as observed in the fermentation fluid of the current study. Many studies have shown that the content of *t*10,*c*12-C18:2 increased with ALA increase in rumen [11], but few have focused on the content of *t*9,*c*12-C18:2. A study reported that ALA supplementation could increase the content of *t*9,*c*12-C18:2 in milk fat [12], and that the milk *t*9,*c*12-C18:2 might originate from the rumen. A previous study reported that *c*12-C18:1, *t*11-C18:1, and *t*9-C18:1 were products of ALA [2,3,4], and *t*11-C18:1 was the main product of ALA biohydrogenation in the rumen [4]. This study also found higher concentrations of *c*12-C18:1, *t*11-C18:1, and *t*9-C18:1 in the fermentation fluid when ALA was added to the fluid, where *t*11-C18:1 increased from 1.804 ng/mL to 3.118 ng/mL at 3 h, *c*12-C18:1 from 0.322 ng/mL to 0.609 ng/mL at 3 h, and *t*9-C18:1 increased from 0.256 ng/mL to 0.404 ng/mL at 5 h.

**Figure 3:**
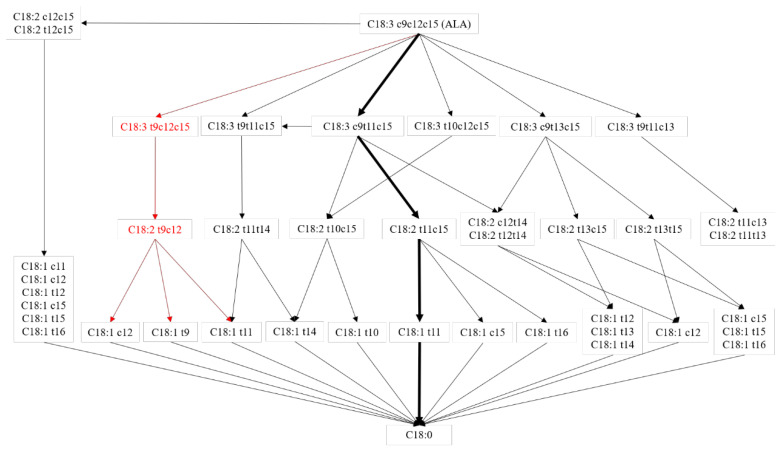
ALA biohydrogenation pathways in the rumen. Adapt from [4]. Red line pathway: reported in this experiment; black line pathway: reported by previous study; black bold line pathway: main biohydrogenation pathways in the rumen.

As shown in Figure 2, with the decreasing concentration of ALA, the content of *t*9,*c*12,*c*15-C18:3 increased in the rumen fermentation fluid, so it was speculated that ALA could convert to *t*9,*c*12,*c*15-C18:3. The concentration of *t*9,*c*12,*c*15-C18:3 was less than 0.048 ng/mL, so the conversion of ALA to *t*9,*c*12,*c*15-C18:3 might not be a major biohydrogenation pathway. A previous study reported that *t*10,*c*12,*c*15-C18:3 could convert to *t*10,*c*15-C18:2 in the rumen [13] and the biohydrogenation occurred in a *cis* double bond. This study showed that ALA supplementation can increase the content of *t*9,*c*12,*c*15-C18:3 and *t*9,*c*12-C18:2 in fermentation fluid, so it is likely that *t*9,*c*12,*c*15-C18:3 could convert to *t*9,*c*12-C18:2, but *t*9,*c*12,*c*15-C18:3 was not the main source of *t*9,*c*12-C18:2, due to the low concentration of *t*9,*c*12,*c*15-C18:3 in fermentation fluid. To the best of our knowledge, no article has reported the next transformation pathway of *t*9,*c*12-C18:2. However, *t*11,*c*15-C18:2 was one of the isomers of *t*9,*c*12-C18:2 reported that *t*11,*c*15-C18:2 could covert to *c*15-C18:1, *t*11-C18:1, and *t*16-C18:1 via *t*11,*c*15-C18:2 to *c*15-C18:1, *t*11,*c*15-C18:2 to *c*15-C18:1 and *t*11,*c*15-C18:2to *t*11-C18:1) [2]. The conclusion was that *c*12-C18:1, *t*11-C18:1, and *t*9-C18:1 might also be products of *t*9*c*12-C18:2 biohydrogenation, and a higher content of *c*12-C18:1, *t*11-C18:1, and *t*9-C18:1 were also observed in ALA group fermentation fluid. The *c*12-C18:1, *t*11-C18:1, and *t*9-C18:1 were further hydrogenated to C18:0 [4]. On the gas chromatography-mass spectrometry analyses above, *t*9,*c*12,*c*15-C18:3 was the biohydrogenation product of ALA in the rumen and the biohydrogenation pathways may exist in the rumen as *t*9,*c*12,*c*15-C18:3 to *t*9,*c*12-C18:2 to *c*12-C18:1/*t*11-C18:1/*t*9-C18:1 to C18:0 as shown by the red line in Figure 3. Numerous studies in vivo and in vitro have reported multiple biohydrogenation pathways for ALA in the rumen [4]. The *t*9,*t*11,c15-C18:3 contains two *trans* configurations, and was previously reported as a biohydrogenation product of ALA, and *t*9,*t*11,c15-C18:3 was converted from *c*9,*t*11,*c*15-C18:3 [14]. The *t*9,*c*12,*c*15-C18:3 might also isomerize to *t*9,*t*11,*c*15-C18:3 in the rumen, but due to the lesser standard of *t*9,*t*11,*c*15 C18:3, this study did not detect it. Further analysis could explore whether *t*9,*c*12,*c*15-C18:3 could convert to *t*9,*t*11,*c*15-C18:3.

## 4. Conclusions

Overall, this study found that *t*9,*c*12,*c*15-C18:3 was the product of ALA biohydrogenation in the rumen, which may provide a new biohydrogenation pathway in the rumen. The *cis-trans* isomerization of ALA from *c*9,*c*12,*c*15 C18:3 isomerizes to *t*9,*c*12,*c*15-C18:3 could happen in situ and the ortho-position *c*9,*c*12,*c*15-C18:3 isomerized to *t*10,*c*12,*c*15-C18:3. The enzymes and microorganisms involved in isomerization may be different, and any further study should focus on the manipulation of enzymes and microorganisms involved in the biohydrogenation pathway.

## Data Availability

The raw data presented in this study are available in Supplementary Materials, Supplementary Table S2.

## Supplementary Materials

The following supporting information can be downloaded at: www.mdpi.com/article/10.3390/ani12040502/s1, Table S1: Composition of diets (% DM basis), Table S2: the raw data presented in this study.
