# Supplementary material for: Biohydrogenation Pathway of α-Linolenic Acid in Rumen of Dairy Cow In Vitro
Source: Animals (Basel). 2022 Feb 17;12(4):502. doi: 10.3390/ani12040502 (PMC8868468; doi:10.3390/ani12040502)
Supplement: Supplementary file 1 [file animals-12-00502-s001.zip › animals-1550273- suplementary- author proofed.pdf]

Supplementation Table S1. Composition of diets (% DM basis).

Items	Concentration (% of DM)
Diet ingredient	
Corn Silage	21.51
Alfalfa hay	8.76
Alfalfa semi-dry silage	3.61
Wrapped straw	2.44
Corn	26.18
Corn flakes	2.77
Wool cotton seed	3.88
Orange peel granule	1.94
Soybean meal	12.42
Puffed soybeans	2.22
Cotton meal	3.12
DDGS	2.85
Bran	1.08
Fat powder	1.71
Premix	5.51
Total	100
Chemical, % of DM	
DM	51.18
NE_L, Mcal/kg of DM	1.91
CP	17.19
ADF	17.99
NDF	63.15
EE	4.59
FA, % of total FA reported	
C14:0	2.04
C16:0	76.05
c9-C16:1	0.03
C18:0	0.03
c9-C18:1	0.77
c9,c12-C18:2	18.86
c9,c12,c15-C18:3	2.10
C20:0	0.04
c11-C20:1	0.01
C22:0	0.04
C24:0	0.03

DM = dry matter; NEL = net energy for lactation; CP = crude protein; ADF = acid detergent fiber; NDF = neutral detergent fiber; EE = ether extracts.
